# Youth and Old Age

**Published:** 1891-10

**Authors:** 


### YOUTH AND OLD AGE.

As Dr. Oliver Wendell Holmes once said, "It is better to be seventy years *young*, than forty years *old*."

Dr. Morell Mackenzie has voiced the same sentiment more at length. In a recent article he says:

Some men are old at forty, while others may be almost said to be young at eighty. A man is just as old as his tissues, particularly those of his heart and brain, and there are octogenarians who, for mental and even physical volatility, might be their own grandsons. The secret of such perpetual youth lies mostly in regular exercise, whether in felling trees or in the humbler form of the daily "constitutional." Even when life has at last fallen into the sere and yellow leaf, exercise of a kind and amount suited to the "shrunk shanks," stiff joints, brittle bones, and other evidences of senility, will keep the furnace of the vital locomotive aglow long after others less carefully stoked have paled their ineffectual fires. But this can only be done (to continue the metaphor)

by slackening speed and reducing pressure. If old men will jump hedges as in their salad days, they will not improbably do so to a musical accompaniment of snapping thigh bones. If they run to catch trains their hearts are extremely likely to mark their sense of such an outrage by stopping work. Dr. Hammond, of Washington, has collected seventy cases which have occurred in that city during the last ten years of men dying suddenly from running after street cars.

If a man has ridden all his life he may continue to do so as long as he can sit on a horse, otherwise this exercise is too violent for the aged. The "constitutional" is unquestionably the sheet anchor of old age as far as exercise is concerned. I need say nothing more about it than that each walk should be taken with a definite purpose, if it is only to set one's watch by a particular clock. To have an object of some kind makes all the difference between wholesome exercise and the listless dragging about of the dead weight of one's own body, which makes walking one of the most fatiguing as well as the dreariest of all forms of motion. To sum up the whole subject, the golden rule for exercise through all the seven chapters of man's strange, uneventful history, is to use it that the stream of life shall flow swift and clear, never stagnating like a muddy pond, and on the other hand, never dashing itself to pieces in mere foam and fury.

